# Bibliographical Notices

**Published:** 1846-09

**Authors:** 


### BIBLIOGRAPHICAL NOTICES.

*Manual of Orthopedic Surgery, being a dissertation which obtained the Boylston prize for* 1844, *by* HENRY JACOB BIGELOW, M. D. Boston, William D. Ticknor & Co. 1845; 8vo. pp. 208.

This work contains the latest and most approved principles and methods appertaining to Orthopedic Surgery, systematically arranged, and presented in a compact and clear manner. The mechanical execution is highly creditable to the Publishers. It is illustrated with several well executed cuts exhibiting the different apparatus employed in this interesting department of surgery.

---

*Anatomy and Physiology designed for Academies and families, by* Calvin Cutler, M. D. *Third stereotype edition, with over two hundred engravings.* Boston, Benjamin B. Mussey; 12mo. pp. 342, 1846.

This work is designed to serve as a text book in academic institutions where Anatomy and Physiology enter into the system of instruction, and for classes who wish to acquire a general knowledge of the mechanism of their own bodies. Of the merits and claims of these sciences, irrespective of their medical relations, we need not here speak. That they are becoming more and more appreciated is shown by the multiplication of popular treatises on the subjects. We have bestowed some examination on this work, and deem it well adapted to fulfil the objects for which it is intended. The rapid succession of editions, and the fact that the publisher now issues a stereotyped edition, are indications of the favor with which it has been received.

---

*Physical Education and the Preservation of Health, by* John C. Warren, M. D. *Prof. of Anat. and Surg. in Harvard University.* Boston, William D. Ticknor & Co. 1846, 12mo. pp. 90.

A few general principles, relating to one of the most important of subjects, by one whose position and reputation must give to his counsels great confidence and force with the public — for whose benefit this little work was written.

---

*Scrofula; its nature, its causes, its prevalence, and the principles of treatment, by* Benjamin Phillips, F. R. S. &c. Philadelphia, Lea & Blanchard, 1846, 8vo. pp. 350.

We have not, as yet, found time to give to this work that degree of close examination to which from its character, and the importance of the subject, it is entitled. The author has evidently bestowed great labor in collecting materials for as full an elucidation of all the circumstances connected with the disease, as the present state of science will admit of. The accumulation of statistical facts is particularly valuable. We shall give an extended notice of the work in our next number.

---

*A practical treatise on the Diseases of Children, by* James Milman Coley, M. D. *Member of the Royal College of Physicians in London, &c. &c.* Philadelphia, Ed. Barrington & Geo. D. Haswell, 1846, pp. 414, 8vo.

This is the title of the Quarterly Volume of the *Select Medical Library*, just issued. The author says in his introduction, "the practical portions have been the result of an experience of forty years, during which time I have been constantly engaged in recording cases, accumulating facts, and pursuing pathological inquiries." In looking over the work we are agreeably impressed with its comprehensive and practical character. It is evidently not a compilation, but, what the author declares it to be, the results of his own long and large experience. We think that the selection of the work by the editor of the Library, Dr. Bell, is a highly judicious one; for, certainly, there is not a more interesting or important department of practical medicine than that relating to diseases of children; nor is there any in which the collection and comparison of individual experience and observation are more eagerly sought after by the practitioner. Again we avail ourselves of an opportunity to commend to the attention of our medical brethren the *Select Medical Library*. For five dollars subscribers receive quarterly a valuable medical work, selected, from among the host which are being issued, by the learned Editor, Dr. Bell; and, in addition, a monthly periodical, the *Bulletin of Medical Science*, which comes regularly, freighted with choice selections and sterling editorials.

